# Effects of Specific Multi-Nutrient Enriched Diets on Cerebral Metabolism, Cognition and Neuropathology in AβPPswe-PS1dE9 Mice

**DOI:** 10.1371/journal.pone.0075393

**Published:** 2013-09-24

**Authors:** Diane Jansen, Valerio Zerbi, Ilse A. C. Arnoldussen, Maximilian Wiesmann, Anne Rijpma, Xiaotian T. Fang, Pieter J. Dederen, Martina P. C. Mutsaers, Laus M. Broersen, Dieter Lütjohann, Malgorzata Miller, Leo A. B. Joosten, Arend Heerschap, Amanda J. Kiliaan

**Affiliations:** 1 Department of Anatomy, Radboud University Nijmegen Medical Centre, Donders Institute for Brain, Cognition and Behaviour, Nijmegen, The Netherlands; 2 Nutricia Advanced Medical Nutrition, Danone Research, Centre for Specialised Nutrition, Wageningen, The Netherlands; 3 Institute for Clinical Chemistry and Clinical Pharmacology, University Clinics Bonn, Bonn, Germany; 4 Department of General Internal Medicine, Radboud University Nijmegen Medical Centre, Nijmegen Institute for Infection, Inflammation and Immunity, Nijmegen, The Netherlands; 5 Department of Radiology, Radboud University Nijmegen Medical Centre, Nijmegen, The Netherlands; Massachusetts General Hospital/Harvard Medical School, United States of America

## Abstract

Recent studies have focused on the use of multi-nutrient dietary interventions in search of alternatives for the treatment and prevention of Alzheimer's disease (AD). In this study we investigated to which extent long-term consumption of two specific multi-nutrient diets can modulate AD-related etiopathogenic mechanisms and behavior in 11-12-month-old AβPPswe-PS1dE9 mice. Starting from 2 months of age, male AβPP-PS1 mice and wild-type littermates were fed either a control diet, the DHA+EPA+UMP (DEU) diet enriched with uridine monophosphate (UMP) and the omega-3 fatty acids docosahexaenoic acid (DHA) and eicosapentaenoic acid (EPA), or the Fortasyn® Connect (FC) diet enriched with the DEU diet plus phospholipids, choline, folic acid, vitamins and antioxidants. We performed behavioral testing, proton magnetic resonance spectroscopy, immunohistochemistry, biochemical analyses and quantitative real-time PCR to gain a better understanding of the potential mechanisms by which these multi-nutrient diets exert protective properties against AD. Our results show that both diets were equally effective in changing brain fatty acid and cholesterol profiles. However, the diets differentially affected AD-related pathologies and behavioral measures, suggesting that the effectiveness of specific nutrients may depend on the dietary context in which they are provided. The FC diet was more effective than the DEU diet in counteracting neurodegenerative aspects of AD and enhancing processes involved in neuronal maintenance and repair. Both diets elevated interleukin-1β mRNA levels in AβPP-PS1 and wild-type mice. The FC diet additionally restored neurogenesis in AβPP-PS1 mice, decreased hippocampal levels of unbound choline-containing compounds in wild-type and AβPP-PS1 animals, suggesting diminished membrane turnover, and decreased anxiety-related behavior in the open field behavior. In conclusion, the current data indicate that specific multi-nutrient diets can influence AD-related etiopathogenic processes. Intervention with the FC diet might be of interest for several other neurodegenerative and neurological disorders.

## Introduction

Alzheimer's disease (AD) is a complex neurodegenerative disorder that affects over 36 million people worldwide. The exact cause of AD is still largely unknown despite over 100 years of extensive research, and still no curative treatments are available. Aging is recognized as the main risk factor for the late-onset sporadic form of AD (SAD), while early-onset familial AD (FAD) has been linked to autosomal dominant mutations in the gene for the amyloid-β precursor protein (AβPP) and the genes for the presenilin 1 (PS1) and presenilin 2 (PS2) proteins [1], [2]. Both SAD and FAD share specific neuropathologic features, including neurofibrillary tangles, amyloid-β (Aβ) plaques, neuronal loss, white matter lesions and synaptic changes in vulnerable brain regions such as the hippocampus and neocortex [3], [4]. For decades, the production and accumulation of the Aβ peptide has been proposed to be the primary trigger of the pathological cascade leading to neurodegeneration and the development of AD. Besides Aβ, several other (risk) factors have been proposed to play an important role in the development of AD.

Many large epidemiological studies have demonstrated that vascular disorders, such as hypercholesterolemia and atherosclerosis, are important risk factors for AD [5]–[7]. Furthermore, cardiovascular disease risk factors, such as a sedentary lifestyle, high saturated fatty acid (SFA) intake, diabetes, smoking and obesity, are associated with a higher risk of developing AD and other dementias [8]–[11]. Many of these cardiovascular risk factors are modifiable. Modifying cardiovascular risk factors, for example by changing lifestyle, might ultimately also affect the risk of developing AD.

Due to the limited and short-lasting efficacy of the current drugs available [12], recent work has focused on the use of dietary interventions for the treatment and prevention of AD. Omega-3 long-chain poly-unsaturated fatty acids (n3 lc-PUFAs), such as docosahexaenoic acid (DHA) and eicosapentaenoic acid (EPA), have shown protective properties with regard to risk of age-related cognitive decline and AD [13]–[16]. The mechanisms by which these dietary nutrients exert protective properties against AD are still under investigation, but several lines of evidence have shown beneficial effects of n3 lc-PUFAs on the cardiovascular system [17], [18] and on neuronal membrane properties [19], [20]. These beneficial effects on the cardiovascular system have been explained by the capacity to decrease blood pressure [21], lower plasma triacylglycerols [22], [23], prevent arrhythmias [24], improve vascular reactivity [25], [26], decrease atherosclerosis [27], and suppress inflammatory processes [28]. Furthermore, high levels of n3 lc-PUFAs replace omega-6 fatty acids (n6 FAs) and cholesterol from cell membranes, leading to increased membrane fluidity, increased number of receptors, enhanced receptor binding and affinity, better ion channel functionality, and modulation of gene expression of many enzyme proteins involved in signal transduction processes [29]–[31]. As a result, this will lead to improved neurotransmission and signaling [32], which is important for optimal cognitive functioning [33]. Other dietary components, like B vitamins and antioxidants, have also been shown to protect the brain from oxidative and inflammatory damage [34]–[36], and synaptic and neuronal loss [37], [38]. However, when tested in a clinical setting supplementation with single nutrients is marginally effective in improving disease status [39]–[42]. It has been suggested that approaches with multiple nutritional components might be more promising, since not individual nutrients but dietary patterns were identified as a factor influencing the risk of developing AD [43].

Combined administration of different nutrients has shown increased effectiveness in altering specific parameters involved in AD. Supplementation with DHA or uridine monophosphate (UMP) improved water maze performance of environmentally impoverished rats. However, the combined administration of DHA and UMP was more effective in improving learning abilities [44]. Furthermore, performance on the four-arm radial maze, T-maze and Y-maze tests by normal adult gerbils was improved by supplementation of DHA and choline, and was even further enhanced by coadministering UMP [45]. In addition to enhancing cognitive performance, combinations of nutrients were shown to be more effective than single nutrients in counteracting neurodegenerative aspects of AD [38], [46], [47] and enhancing processes involved in neuronal regeneration and function [48]–[50]. Moreover, clinical trials with combinations of nutrients have shown beneficial effects on memory performance in patients with mild AD [51]–[53].

In the current study, we wanted to investigate the extent to which long-term consumption of two specific multi-nutrients diets can modulate behavior, cognition, hippocampal metabolite levels, neurogenesis and inflammation in 11-12-month-old AβPP-PS1 mice. Starting from 2 months of age, animals were fed either a control diet, a multi-nutrient diet enriched with DHA, EPA and UMP (DEU), or a multi-nutrient diet enriched with DHA, EPA, UMP, phospholipids, choline, folic acid, vitamins B6, B12, C,E and selenium (Fortasyn® Connect). We performed behavioral testing, proton magnetic resonance spectroscopy (^1^H MRS), immunohistochemistry, biochemical analyses and quantitative real-time PCR to gain a better understanding of the potential mechanisms by which these multi-nutrient diets may exert protective properties against AD.

## Animals, Materials and Methods

### Ethics statement, animals and housing conditions

The experiments were performed according to Dutch federal regulations for animal protection and were approved by the Veterinary Authority of the Radboud University Nijmegen Medical Centre (Permit Number: RU-DEC 2008-126h). All efforts were made to minimize suffering of the animals.

The AβPPswe-PS1dE9 founders were originally obtained from Johns Hopkins University, Baltimore, MD, USA (D. Borchelt and J. Jankowsky, Dept. of Pathology) and a colony was established at the Radboud University Nijmegen Medical Centre, The Netherlands. In short, mice were created by co-injection of chimeric mouse/human AβPPswe (mouse AβPP695 harboring a human Aβ domain and mutations K595N and M596L linked to Swedish familial AD pedigrees) and human PS1dE9 (deletion of exon 9) vectors controlled by independent mouse prion protein promoter elements. The two transfected genes co-integrate and co-segregate as a single locus [54], [55]. This line (line 85) was originally maintained on a hybrid background by backcrossing to C3HeJ × C57BL6/J F1 mice (so-called pseudo F2 stage). For the present work, the breeder mice were backcrossed to C57BL6/J for 13 generations to obtain mice for the current study. Throughout the experiment, animals were housed in groups of 2–6 mice per cage in a controlled environment, homogenously illuminated by normal fluorescent room light at 60 lux, with room temperature at 21°C, and an artificial 12∶12 h light:dark cycle (lights on at 7 a.m.). Food and water were available *ad libitum*.

**Figure 1:**
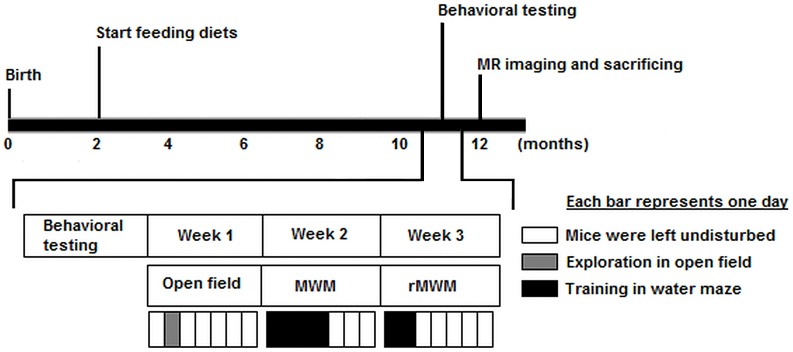
Time line of experimental design. At 2 months of age, AβPP-PS1 mice and their wild-type littermates were put on either a control diet (CO), a DHA, EPA and UMP diet (DEU) or a Fortasyn® Connect diet (FC) for the remainder of the experiment. Behavioral testing was performed at 11 months of age. Animals were weight one week before starting the behavioral testing battery. In the first week of behavioral testing, animals were exposed to the open field once for 30 minutes. In the second week, animals were trained in the Morris water maze (MWM) for 4 days. In the third week, animals were trained in the reverse Morris water maze (rMWM) for 2 days. MR imaging was performed at 12 months of age. Mice were weighed before MR scanning and sacrificed immediately after MR imaging.

Male transgenic AβPP-PS1 mice and their wild-type littermates were fed either 1] a standard Control diet (CO diet), 2] a multi-nutrient diet enriched with DHA, EPA and UMP (DEU diet), or 3] a multi-nutrient diet, called Fortasyn® Connect (FC diet), containing precursors and cofactors in membrane synthesis and maintenance via the Kennedy cycle [56], such as DHA, EPA, UMP, phospholipids, choline, vitamins B6, B9, B12, C and E, folic acid and selenium. The diets differed in composition with regard to the fat blends used, as well as a number of supplemented nutrients as indicated in Table 1. Diets were isocaloric and were manufactured by Research Diet Services (Wijk bij Duurstede, The Netherlands). In order to minimize oxidation of the n3 lc-PUFAs, the experimental diets were stored at −20°C in 2-day supply aliquots. Feeding the diets started when the mice reached the age of 2 months and was maintained for the remainder of the experiment. Animals underwent behavioral testing at 11 months of age and subsequently MRI measurements at 12 months of age (Figure 1). In total, 85 mice were used in the current study. Table 2 describes the number of mice used in each experimental group. The body weight of the mice was determined one week before the start of the behavioral tests at 11 months of age, and again on the day of the MRI measurements at 12 months of age.

**Table 1:** Compositions of the experimental diets used, based on AIN-93M [172] with minor revisions.

	Dietary groups		
**Source**	Control (CO)	DHA, EPA, UMP (DEU)	Fortasyn® Connect (FC)
	g/100 g of diet	g/100 g of diet	g/100 g of diet
Corn Starch	35.57	34.57	33.12
Casein (>85% protein)	14.00	14.00	14.00
Corn dextrin	15.50	15.50	15.50
Sucrose	10.00	10.00	10.00
Dextrose	10.00	10.00	10.00
Fibers	5.00	5.00	5.00
Mineral mix (AIN-93M-MX)	3.50	3.50	3.50
Vitamin mix (AIN-93-VX)	1.00	1.00	1.00
**Fats**			
Soy oil	1.900	–	–
Coconut oil	0.900	0.100	0.100
Corn oil	2.200	1.870	1.870
Fish oil	–	3.030	3.030
**Additions**			
L-cysteine	0.180	0.180	0.180
Choline bitartrate (41.1% choline)	0.250	0.250	0.250
Tert-butylhydroquinone	0.0008	0.0008	0.0008
Pyridoxine-HCl	–	–	0.00328
Folic acid (90%)	–	–	0.00067
Cyanocobalamin (0.1% in mannitol)	–	–	0.00350
Ascorbic acid (100% pure)	–	–	0.160
dl-α-tocopheryl acetate (500 IU/g)	–	–	0.4650
UMP disodium (24% H_2_O)	–	1.0	1.0
Choline chloride (74.576%)	–	–	0.402
Soy lecithin	–	–	0.402
Sodium selenite (46% min)	–	–	0.00023
**Energy (kcal/100** **g chow)**	**376.9**	**372.9**	**367.1**

All diets were isoenergetic, contained 5% fat and standard vitamin and mineral premix, providing recommended daily amounts of these nutrients. DHA = docosahexaenoic acid; EPA = eicosapentaenoic acid; UMP = uridine monophosphate.

**Table 2:** Overview of the number of mice used in each experimental group.

Genotype	Diet	Total	OF	(r)MWM	MRS	IHC	BCH
Wild-type	CO	20	20	20	13–15	15	9
	DEU	17	17	17	13–15	8	8
	FC	16	16	16	14–15	16	7
AβPP-PS1	CO	14	14	13	10	5–7	5
	DEU	8	8	8	7	4–5	3
	FC	10	10	10	7	4–6	4

CO = Control diet; DEU =  DHA, EPA, UMP diet; FC = Fortasyn® Connect diet; OF = open field; (r)MWM = (reverse) Morris water maze; MRS = magnetic resonance spectroscopy; IHC = immunohistochemistry; BCH = biochemistry.

### Behavioral analyses

Behavioral testing was performed in the following order (Figure 1): First open field, followed by the Morris water maze (MWM), and finally the reversal MWM (rMWM). All testing sessions were performed during the light phase (between 9 a.m. and 5 p.m.) and were recorded for computer-assisted analysis using Ethovision XT 7.0 software (Noldus Information Technology B.V., Wageningen, The Netherlands). All behavioral testing was performed in the same room, homogenously illuminated by normal fluorescent room light at 60 lux.

#### Open field

To analyze explorative and anxiety-related behavior, mice were placed individually in the center of a square open field (50×50×40 cm) with white Plexiglas walls. Animals were observed for 30 minutes, and the durations (seconds) of walking, wall leaning, rearing, sitting and grooming were scored and analyzed. These open field parameters were defined as described previously [57], [58]. In addition, total walking distance, mean velocity, and the time spent in the corners and in the center of the open field were obtained from the recorded sessions. The center of the open field was defined as a square measuring 20×20 cm, and the corners of the open field were defined as the sum of all four 10×10 cm squared corners.

#### Morris water maze (MWM)

To investigate spatial learning abilities, mice were tested in the Morris water maze (MWM). In short, mice were placed in a pool (104 cm diameter) at different starting positions and were trained to find a submerged platform by using distant visual cues in the room. The water was made opaque by the addition of milk powder, and was kept at a constant temperature of 21-22°C. The maze was surrounded by white curtains at a distance of 0.5 meter, which were marked by four spatial cues varying in shape, size and color. The 8 cm diameter round platform was submerged 1 cm below the water surface and was placed in the middle of the northeast (NE) quadrant at a distance of approximately 26 cm from the wall. The researcher was always present at the same location in the room during all trials (close to the southwest quadrant.

Acquisition (spatial learning): Mice were trained to find the location of the submerged escape platform in 4 acquisition trials per day (maximal swimming time 120 s; 30 s on the platform; inter-trial interval 60 min) during 4 consecutive days. The latency time (s) to find the hidden platform was scored. Starting positions during the 4 trails/day were: south (S), north (N), east (E), west (W). After each trial, mice were placed back in their home cage, and a paper towel was available inside the cage for additional drying.

Probe (spatial memory): All mice performed a single probe trial 60 min after the last trial on day 4. The platform was removed from the swimming pool and mice were allowed to swim for 120 s. The time spent swimming and searching in the NE quadrant (former platform quadrant), the total swimming distance, the mean velocity and the time spent swimming at the exact former platform location were measured.

#### Reverse Morris water maze (rMWM)

Four days after the standard MWM probe trial, a simplified reversal MWM [58], [59] was performed in which the platform was relocated to a new position in the southwest (SW) quadrant of the pool. In this procedure, memory retrieval needs to be selective for the most recently learned location, introducing an episodic like component in the spatial memory task [60]. Acquisition and probe sessions were performed similarly to the standard MWM sessions, except that starting positions were E, W, S, and N, the target quadrant was SW, and training lasted only 2 days (4 trials/day).

### Magnetic resonance imaging (MRI)

MRI measurements were performed on a 11.7T BioSpec Avance III small animal MR system (Bruker Biospin, Ettlingen, Germany) equipped with an actively shielded gradient set of 600 mT/m. A circular polarized volume resonator was used for signal transmission and an actively decoupled mouse brain quadrature surface coil was used for signal detection (Bruker BioSpin). During the experiments, mice were anesthetized with 3.5% isoflurane (Nicholas Primal (I) limited, London, United Kingdom) for induction and 2% isoflurane for maintenance in a mixture of N_2_O and oxygen (1∶2) through a nose cone. The anesthetic concentration was adjusted during the experiment in order to maintain the breathing frequency at 70–100 per minute. Respiration of the animal was monitored using a pneumatic cushion respiratory monitoring system (Small Animal Instruments Inc., NY, USA). Body temperature was measured using a rectal thermometer and maintained at 37°C using a heated air flow device. Mice were placed in a stereotactic holder in order to immobilize the head and prevent unwanted movement during the scanning. Gradient echo (GE) images in the axial, sagittal and coronal orientation were acquired to visualize the anatomy of the mouse brain structures. Imaging parameters were: echo time (TE) = 5 ms, repetition time (TR) = 630 ms, flip angle = 12 deg, field of view (FOV) = 40×40 mm, matrix size = 512×512, slice thickness = 0.345 mm.

#### Magnetic resonance spectroscopy (MRS)

Metabolite concentrations in the hippocampus were determined using ^1^H MRS with single voxel technique. The spectroscopic volume of interest (VOI) of 1.0×1.0×2.0 mm was positioned unilaterally in the right hippocampus based on the acquired anatomical images. Water-suppressed ^1^H-MRS spectra were acquired with a point-resolved spectroscopy sequence (PRESS) with a short echo time with imaging parameters: TE = 10.905 ms, TR = 2500 ms, T1 = 6.31 ms, T2 = 4.59 ms, and 800 signal averages. Total acquisition time for ^1^H MRS was 27 min per animal.

Quantification of the metabolite concentration was performed using a the Linear Combination (LC) model software package (LCModel™, S. Provencher, Oakville, Canada). The quantification algorithm of LCModel™applies linear combinations of model spectra to calculate the best fit of the experimental spectrum. The model spectra (dataset of prior knowledge) were calibrated to match the magnetic field strength, sequence type and sequence parameters used for data acquisition.

The criteria to select reliable metabolite tissue concentrations were based on the Cramér-Rao lower bounds (CRLB), which are estimates of the S.D. of the fit for each metabolite [61] as determined by LCModel™. Only CRLB≤20% were included in the analysis. Concentrations with CRLB>20% were classified as not detected. Six metabolites fulfilled the criteria: choline + glycerophosphocholine + phosphocholine (tCho; choline-containing compounds), creatine + phosphocreatine (tCre), glutamine + glutamate (Glx), *myo*-Inositol + glycine (*m*I+Gly), *N*-acetylaspartate + *N*-acetylaspartylglutamate (tNAA) and taurine (Tau). Although the exact functions of these metabolites are not fully known, tNAA is considered to be a marker of neuronal viability, tCre is involved in energy metabolism, *m*I is a putative marker for microglia and astrogliosis, and tCho is required for the synthesis of the neurotransmitter acetylcholine, and of phosphatidylcholine, a major constituent of membranes, and is therefore associated with membrane turnover [62].

### Tissue sampling

Directly following the MR measurements at 12 months of age, half of the number of mice was sacrificed by cervical dislocation to collect blood samples and brain tissue for biochemical analyses, and the other half was sacrificed by transcardial perfusion fixation with 4% paraformaldehyde (4% paraformaldehyde in 0.1 M phosphate buffered saline (PBS; pH = 7.3) to collect brains for immunohistochemical stainings. Blood samples were collected via eye extraction, and subsequently processed to obtain blood serum. Blood serum was stored at -80°C, before further biochemical processing. Non-perfused brains were snap frozen in liquid nitrogen and then stored at −80°C, before further biochemical processing. Perfused brains were collected and postfixed for 15 h at 4°C in 4% paraformaldehyde fixative and subsequently stored in 0.1 M PBS with 0.01% sodium azide at 4°C before further immunohistochemical processing.

### Immunohistochemistry

Before cutting, the brain tissue was cryoprotected by immersion in 30% sucrose in 0.1 M PBS. Six series of 40 µm coronal sections were cut through the brain using a sliding microtome (Microm HM 440 E, Walldorf, Germany) equipped with an object table for freeze sectioning at −60°C.

Immunohistochemistry was performed using standard free-floating labeling procedures, and was carried out on a shaker table at room temperature.

#### Doublecortin staining

Immature neurons were visualized with anti-doublecortin antibody (polyclonal goat anti-doublecortin (C18): sc-8066, Santa Cruz Biotechnology, Inc., Santa Cruz, CA, USA) using one complete subseries of brain sections per animal with 240 µm distance between the sections. Doublecortin is a microtubule-associated protein that is exclusively found in somata and processes of migrating and differentiating neurons [63], [64]. In short, after blocking the brain sections against endogenous peroxidase with 0.3% H_2_O_2_ in 0.1 M PBS for 30 minutes, the sections were pre-incubated with PBS-BT (0.1 M PBS with 0.1% Bovine Serum Albumin and 0.3% Triton-X-100) for 30 minutes. Brain sections were incubated overnight with polyclonal goat anti-doublecortin (1∶3000 diluted in PBS-BT) as primary antibody. After incubating for 90 minutes with donkey anti-goat biotin secondary antibody (1∶1500 diluted in PBS-BT, Jackson ImmunoResearch, West Grove, PA, USA), sections were transferred to a solution containing Vector ABC-elite (1∶800 in PBS-BT; Vector Laboratories, Burlingame, CA, USA) for another 90 minutes. Visualization of doublecortin-positive cells was achieved by incubation with 0.02% 3-3′diaminobenzidin tetra hydrochloride with 0.3% ammonium nickel sulphate as an intensifier in 0.05 M Tris buffer (DAB-Ni solution, pH = 7.6) with 0.006% H_2_O_2_ for 10 min. After washing with 0.1 M PBS, all stained sections were mounted on gelatin-coated slides (0.5% gelatin and 0.05% chrome-alum), dried overnight in a stove at 37°C, dehydrated in alcohol series, cleared with xylol and mounted in Entellan.

#### Quantification

Quantification of the doublecortin-positive newly formed immature neurons in the subgranular zone of the hippocampus was performed using a Zeiss Axioskop microscope equipped with hardware and software from Microbrightfield (Williston, VT, USA). Appropriate sections were digitized using a computer-assisted analysis system (Stereo Investigator). Three sections per animals were used at −.70, −2.18 and −2.46 posterior to bregma, based on the mouse brain atlas of Franklin and Paxinos [65]. Contours were drawn along the borders of the hippocampus at 2.5× magnification and doublecortin-positive (Dcx+) cells were counted at 20× magnification. All quantifications were performed by two independent raters who were blind to the experiment groups. Measurements were averaged to obtain a single value per animal.

### Biochemical analyses

#### Serum and brain sterol analysis

Serum cholesterol levels and the cholesterol precursor lathosterol and its oxidative brain specific metabolites, 24S-hydroxycholesterol and 27-hydroxycholesterol, were measured by gas-chromatography-mass-spectrometry-selected ionmonitoring (GC-MS-SIM) as described in detail previously [66]–[68]. Brains were homogenized and sterols were extracted overnight by chloroform/methanol trimethylsilylated prior to GC-MS-SIM analysis [66], [67].

#### Brain fatty acid analysis

Fatty acid analyses were performed with a part of the brain homogenate (described above). Total lipid was extracted from brain homogenates by methanol and chloroform. Subsequently, samples were centrifuged at 3000 rpm for 10 min and the lower phase (chloroform and lipids) was removed. Chloroform was added to the upper phase, samples were centrifuged again at 3000 rpm for 10 min and the lower phase was combined with the first one. The chloroform fractions were dried in a SpeedVac® and 2 ml methanol and 40 µl concentrated sulfuric acid were added to the dried extract. The samples were heated at 100°C for 60 min, and 2 ml hexane and 0.5 ml 2.5 M sodium hydroxide solution were added. After vortexing and centrifuging the samples for 5 min at 3000 rpm, the upper layer was collected and evaporated in a SpeedVac®. The fatty acids (FAs) were dissolved in 125 µl iso-octane and analyzed on a GC-FID with a CP-SIL88 column (50 m×0.25 mm id. 0.22 film thickness). The n6/n3 ratio was calculated as the sum of analyzed n6 FAs divided by the sum of n3 FA.

#### Quantitative real-time PCR (qRT-PCR)

A part of the frozen brain tissue, including both hippocampi, were collected in 1 ml cold Trizol (Invitrogen, Paisley, UK) and homogenized by sonification. After chloroform extraction and isopropyl alcohol precipitation, RNA was dissolved in 25 µl RNase-free DEPC-treated water. The RNA concentrations were measured with a Nanodrop 1000 spectrophotometer (Thermo Fisher Scientific Inc, Wilmington, DE, USA). cDNA synthesis was performed using 1 µg RNA dissolved in 10 µl RNase-free DEPC-treated water containing 2 µl 5× iScript reaction mix and 0.5 µl iScript reverse transcriptase (iScript cDNA synthesis kit, Bio-Rad Laboratories B.V., Veenendaal, The Netherlands) at 25°C for 5 min, at 42°C for 30 min and at 85°C for 5 min (Eppendorf Thermoblock Mastercycler 5330).

Quantitative real-time PCR (qRT-PCR) was performed in a total volume of 10 µl buffer solution containing 2 µl of template cDNA, 5 µl 2× SYBR Green Master mix (Applied Biosystems, Foster City, CA, USA), 2.92 µl RNase-free DEPC-treated water and 0.04 µl of each primer (100 µM). Primers for cluster of differentiation 36 (CD36), glyceraldehyde-3-phosphate dehydrogenase (GAPDH), interleukin-1β (IL-1β), interleukin-6 (IL-6), monocyte chemoattractant protein 1 (MCP-1) and tumor necrosis factor-α (TNF-α) were designed using Vector Primer Express software (Applied Biosystems). Primer pairs were as follows:

CD36: 5′- ATGGGCTGTGATCGGAACTG-3′ and 5′-GTCTTCCCAATAAGCATGTCTCC-3′; GAPDH: 5′-AGGTCGGTGTGAACGGATTTG-3′ and 5′-TGTAGACCATGTAGTTGAGGTCA-3′;

IL-1β: 5′-GCAACTGTTCCTGAACTCAACT-3′ and 5′-ATCTTTTGGGGTCCGTCAACT-3′; IL-6: 5′-CAAGTCGGAGGCTTAATTACACATG-3′ and 5′-ATTGCCATTGCACAACTCTTTTCT-3′; MCP-1: 5′-CCCAATGAGTAGGCTGGAGA-3′ and 5′-TCTGGACCCATTCCTTCTTG-3′; and TNF-α: 5′-CAGACCCTCACACTCAGATCATCT-3′ and 5′-CCTCCACTTGGTCCTTTGCTA-3′. The optimal temperature cycling protocol was determined to be 95°C for 10 min followed by 40 reaction cycles at 90°C for 15 s and at 60°C for 1 min, using a StepOnePlus real time PCR system (Applied Biosystems, Foster City, CA, USA). The absolute quantities were determined using standard curves, and the validity of the results was checked by running appropriate negative controls. The quantity of cDNA was calculated for each sample with StepOne Software version 2.2.2. Relative gene expression ratios, calculated according to the comparative C_T_ method (also referred to as the 2^−ΔCT^ method) [69], were used to evaluate differences. Relative C_T_ (ΔC_T_) values were calculated by subtracting the C_T_ value of the housekeeping gene GADPH from the C_T_ values of CD36, IL-1β, IL-6, MCP-1 or TNF-α. For each primer, two independent qRT-PCR runs were performed, and the means of their relative values were used for statistical analysis.

### Statistical analysis

Data are expressed as mean ± SEM and were analyzed with SPSS for windows 18.0 software (SPSS Inc. Chicago, IL, USA). The repeated measures ANOVA was used for the acquisition phase of the MWM and rMWM (with the repeated measure: trial block), followed by a Bonferroni post hoc to analyze possible interactions between trial block, genotype and/or diet. If interactions between trial block, genotype and/or diet (between-group-factors) were present, the data were split for the concerning factor and thereafter analyzed again with the repeated measures ANOVA. Multivariate ANOVA's (MANOVAs) were conducted with between group factors: genotype and diet, to analyze possible differences between wild-type and AβPP-PS1 mice and the different diet groups in the open field test, the probe trials of the MWM and rMWM, the body weight and brain weight, hippocampal metabolite concentrations, the amount of immature neurons, and the biochemical analyses. If interactions between genotype and diet (between-group-factors) were present, the data were split for the concerning factor and thereafter analyzed again with the MANOVA. If no interactions between the genotype and diet were present and overall analysis revealed a significant effect of diet, the separate diet groups were analyzed post hoc by using Tukey's HSD test. For clarity reasons, F-values are not displayed. Furthermore, only between-group interactions that reached statistical significance are specified in detail. Statistical significance was set at *p*<0.05.

## Results

### Body and brain weight

All mice were weighed one week before starting the behavioral test battery and again on the day of the MR measurements (1–2 months later). Since body weights within the groups did not change significantly between those two time points (ANOVA, *p*>0.50), the mean weight was used for further statistical analyses. Body weight was affected by genotype (ANOVA, *p*<0.001), but not by dietary intake (ANOVA, *p* = 0.913). No significant genotype×diet interaction was observed (ANOVA, *p* = 0.164). Overall mean body weight was 39.6±0.7 g in the AβPP-PS1 mice and 35.2±0.6 g in the wild-type mice. Absolute brain weight was not affected by genotype (ANOVA, *p* = 0.733) or by diet (ANOVA, *p* = 0.543). No significant genotype×diet interaction was observed (ANOVA, *p* = 0.078). Overall mean brain weight was 0.53±0.00 g in AβPP-PS1 animals and 0.53±0.01 g in wild-type mice.

### Behavioral analyses

#### Open field

In the open field, locomotor activity and active exploration parameters (walking, sitting, wall leaning, rearing) and grooming were scored for 30 minutes. In addition, total walking distance, mean walking velocity, and the time spent in the corners respectively the center of the open field were obtained from the recorded sessions.

**Figure 2:**
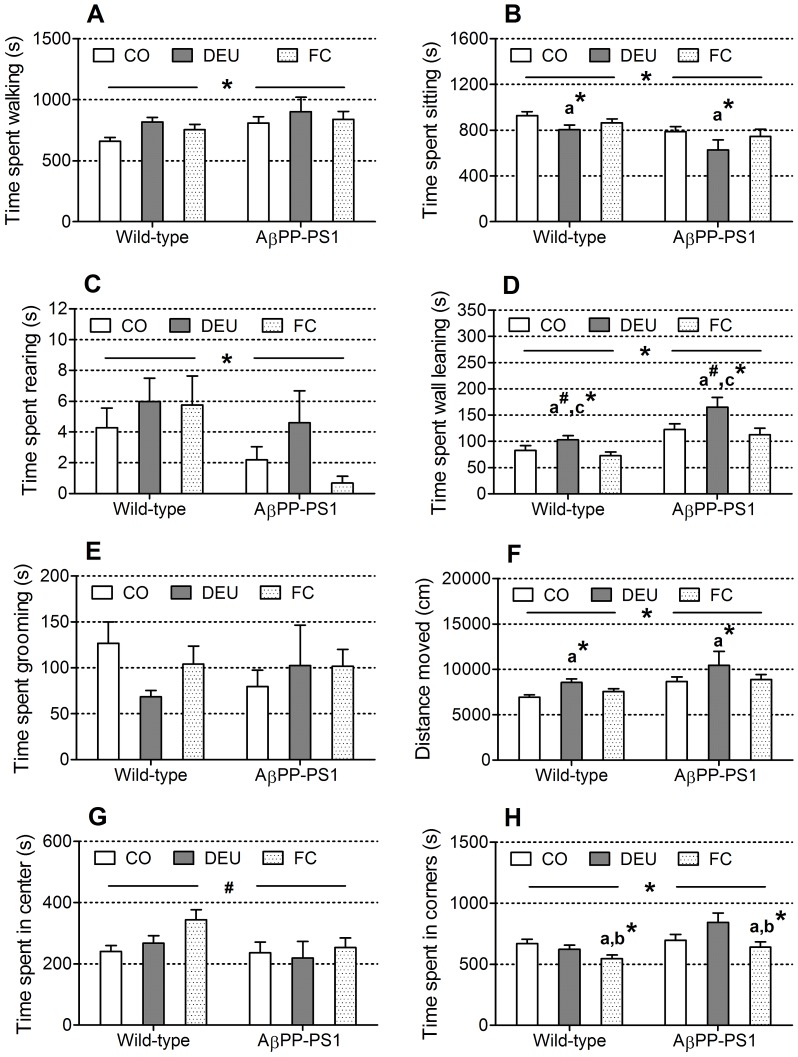
Open field behavior of 11-month-old AβPP-PS1 and wild-type mice on control and specific multi-nutrient diets. Open field parameters were measured within a 30: AβPP-PS1 mice spent more time walking than wild-type mice (*p = 0.015). Walking was not affected by dietary intake. B: AβPP-PS1 mice spent less time sitting than wild-type mice (*p<0.001). Animals on the DHA, EPA and UMP (DEU) diet sat let than animals on control (CO) diet (a* *p* = 0.025), but did not differ from the mice on the Fortasyn® Connect (FC) diet. C: AβPP-PS1 mice spent less time rearing than wild-type mice (*p = 0.030). Rearing behavior was not affected by dietary intake. D: AβPP-PS1 mice spent more time wall leaning than wild-type mice (*p<0.001). Animals fed the DEU diet leaned more against the walls than mice fed the FC diet (c* *p* = 0.007), and also slightly more than animals fed the CO diet (a^#^ *p* = 0.070), although this did not reach statistical significance. E: Grooming behavior was similar among wild-type and AβPP-PS1 mice, and was not affected by dietary intake. F: AβPP-PS1 mice traveled a longer distance than wild-type mice (*p<0.001). Mice fed the DEU diet traveled a greater distance than animals fed the CO diet (a* *p* = 0.009), but did not differ from the animals fed the FC diet. G: AβPP-PS1 mice spent slightly less time in the center of the open field than wild-type mice (^#^ *p* = 0.064), although this did not reach statistical significance. The time spent in the center of the open field was not affected by dietary intake. H: AβPP-PS1 mice spent more time in the corners of the open field compared to wild-type mice (*p = 0.001). Animals fed the FC diet spent less time in the corners of the open field than mice fed the CO diet (a* *p* = 0.038) and animals fed the DEU diet (b* *p* = 0.033).

AβPP-PS1 mice were more active in the open field than wild-type mice, independent of diet (genotype×diet interaction, walking *p* = 0.777; sitting *p* = 0.857; distance moved *p* = 0.926; velocity *p* = 0.927). AβPP-PS1 mice walked more (Figure 2A; ANOVA *p* = 0.015) and sat less (Figure 2B; ANOVA *p*<0.001) than wild-type mice. This resulted in an increased distance moved (Figure 2F; ANOVA *p*<0.001) and higher mean walking speed in AβPP-PS1 mice compared to wild-type animals (ANOVA *p*<0.001; wild-type 4.2±0.1 cm/s, AβPP-PS1 5.1±0.3 cm/s). Dietary intake affected the time spent sitting (ANOVA, *p* = 0.015), the distance moved (ANOVA, *p* = 0.006) and the mean velocity (ANOVA, *p* = 0.006). Dietary intervention with the FC diet had no effect on these parameters (post hoc, *p*>0.05). However, animals fed the DEU diet sat less than animals fed the CO diet (Figure 2B; post hoc *p* = 0.025), but did not differ from the mice fed the FC diet (post hoc, *p* = 0.322). Furthermore, mice fed the DEU diet also traveled a greater distance (Figure 2F; post hoc *p* = 0.009) with a higher mean velocity (post hoc, *p* = 0.009; CO diet 4.2±0.2 cm/s, DEU diet 5.1±0.3 cm/s, FC diet 4.5±0.2 cm/s) than animals fed the CO diet, but again did not differ from the animals fed the FC diet (post hoc, *p* = 0.098). Overall analysis revealed a tendency for a diet effect on the time spent walking (*p* = 0.053), but since this did not reach statistical significance, no additional post hoc tests were performed to compare the different diet groups.

Moreover, AβPP-PS1 mice displayed less explorative behavior away from the walls of the open field, e.g. rearing (Figure 2C; ANOVA *p* = 0.030) compared to wild-type animals, but more explorative behavior against the walls of the open field, e.g. wall leaning (Figure 2D; ANOVA *p*<0.001), independent of diet (genotype×diet interaction, rearing *p* = 0.468; wall leaning *p* = 0.513). Rearing behavior was not affected by dietary intervention with the DEU and FC diets (ANOVA, *p* = 0.355). Wall leaning behavior however was affected by diet (ANOVA, *p* = 0.001). Animals fed the DEU diet leaned more against the walls of the open field than mice fed the FC diet (Figure 2D; post hoc *p* = 0.007), and also slightly more than animals fed the CO diet (post hoc, *p* = 0.070), although this did not reach statistical significance. No significant differences between wild-type and AβPP-PS1 mice (Figure 2E; ANOVA *p* = 0.744) nor between the dietary groups (ANOVA, *p* = 0.620) were observed for grooming behavior (genotype×diet interaction, *p* = 0.172).

Furthermore, AβPP-PS1 mice spent slightly less time in the center of the open field than wild-type mice (Figure 2G; ANOVA *p* = 0.064), although it did not reach statistical significance. No differences were observed between the dietary groups in the time spent in the center of the open field (ANOVA, *p* = 0.116). No genotype×diet interaction was observed for the time spent in the center of the open field (*p* = 0.356). AβPP-PS1 mice did however spent significantly more time in the corners of the open field compared to wild-type animals (Figure 2H; ANOVA *p* = 0.001), independent of diet (genotype×diet interaction, *p* = 0.085). Dietary intake affected the time spent in the corners of the open field (ANOVA, *p* = 0.007), such that animals fed the FC diet spent significantly less time in the corners of the open field than mice fed the CO diet (post hoc, *p* = 0.038) and the DEU diet (post hoc, *p* = 0.033).

Altogether these data show increased activity, but decreased explorative behavior in AβPP-PS1 mice. Furthermore, AβPP-PS1 mice also display increased anxiety-related behavior, as indicated by the increased time spent wall leaning and increased time spent in the corners of the open field [58], [70]. Dietary intervention with the DEU diet increased general locomotor activity and anxiety-related exploration (e.g. wall leaning) in wild-type and AβPP-PS1 mice. Our results might suggest that the FC diet could have an anxiolytic effect, since it decreased the time spent in the corners of the open field in both wild-type and AβPP-PS1 mice.

#### Morris water maze (MWM)

The Morris water maze is designed to test spatial learning by training the mice to find a hidden platform (acquisition phase) in a pool. Spatial memory is tested in a trial in which the platform is removed from the maze (probe trial) following the last trial of the acquisition phase.

**Figure 3:**
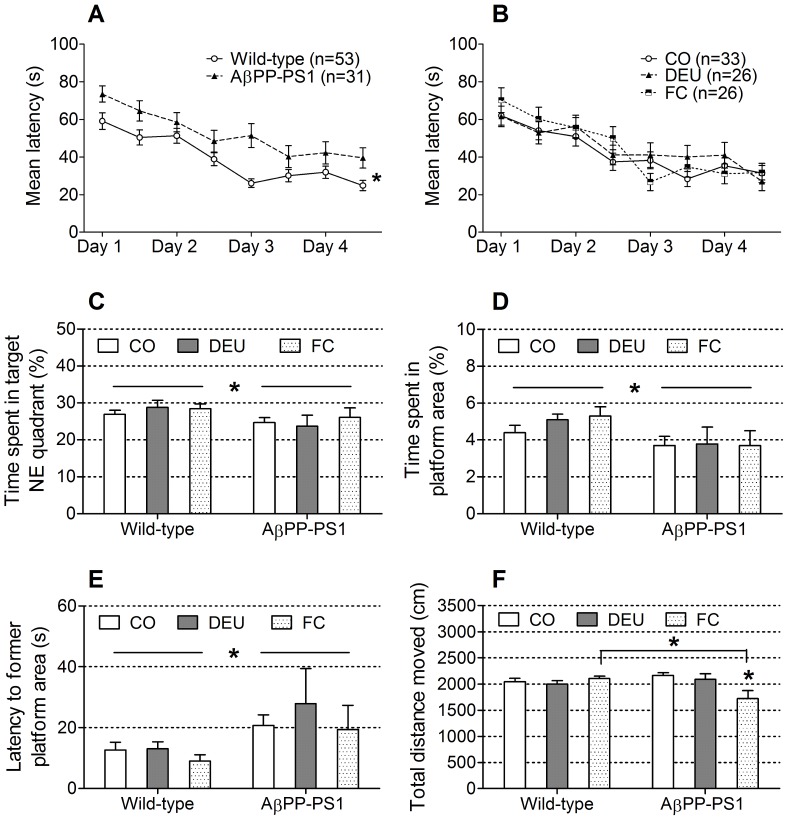
Morris water maze performance of 11-month-old AβPP-PS1 and wild-type mice on control and multi-nutrient diets. Spatial learning was measured in a 4-day acquisition phase, by determining the latency to find a hidden platform in the NE quadrant. Spatial memory was tested in the probe phase in which the percentage of time spent in the target NE quadrant, the time spent in the former platform area, the latency to the former platform area and the total distance moved were measured. A: Both AβPP-PS1 and wild-type mice showed a decrease in latency during training. Overall escape latencies were higher in AβPP-PS1 mice (*p<0.001), independent of dietary intake. B: Animals on control (CO), the DHA, EPA and UMP (DEU) and Fortasyn® Connect (FC) diets showed a similar decrease in latency during training, independent of genotype. Overall escape latencies were not affected by dietary intake. C: AβPP-PS1 mice spent less time in the target NE quadrant than wild-type mice, independent of dietary intake (*p = 0.033). Time spent in the NE quadrant was unaffected by dietary intake. D: AβPP-PS1 mice spent less time in the former platform area than wild-type mice (*p = 0.010), independent of dietary intake. Time spent in the exact former platform area was unaffected by dietary intake. E: AβPP-PS1 mice had a higher latency to reach the former platform location than wild-type mice (*p = 0.003), independent of dietary intake. Latency to reach the former platform location was unaffected by dietary intake. F: AβPP-PS1 mice on FC diet displayed a shorter swim distance compared to wild-type mice on FC diet (*p = 0.009) and compared to AβPP-PS1 mice on CO diet (*p = 0.013), but not compared to AβPP-PS1 mice on DEU diet.

Both AβPP-PS1 and wild-type mice showed a decrease in escape latency during training (Figure 3A; ANOVA *p*<0.001). No significant time×genotype, time×diet or time×genotype×diet interactions were observed (*p*>0.05), indicating that all animals learned the position of the hidden platform equally well. However, escape latencies were significantly higher in AβPP-PS1 mice compared to wild-type animals (Figure 3A; ANOVA *p*<0.001), independent of dietary intake (genotype×diet interaction, *p* = 0.097), indicating that spatial learning was affected by genotype. Escape latencies did not differ between the animals fed the CO diet, DEU diet or FC diet (Figure 3B; ANOVA *p* = 0.203). During acquisition training, swim speed decreased in both AβPP-PS1 and wild-type mice over time (data not shown; ANOVA *p*<0.001). No significant time×genotype, time×diet or time×genotype×diet interactions were observed (*p*>0.05). However, the average swim speed was significantly higher in AβPP-PS1 mice compared to wild-type animals during training (ANOVA *p* = 0.001; wild-type 10.5±0.3 cm/s; AβPP-PS1 12.5±0.5 cm/s), independent of dietary intake (genotype×diet interaction *p* = 0.652). The mean swim speed did not differ (ANOVA, *p* = 0.341) between the animals fed the CO diet (11.5±0.4 cm/s), DEU diet (10.6±0.4 cm/s) or FC diet (11.5±0.6 cm/s).

During the probe trial, AβPP-PS1 mice spent less time in the target NE quadrant (Figure 3C, ANOVA *p* = 0.033) than wild-type mice, independent of diet (genotype×diet interaction, *p* = 0.688), indicating impaired spatial memory. Furthermore, AβPP-PS1 mice also spent less time in the exact area where the platform had been located (Figure 3D; ANOVA *p* = 0.010) and had a higher latency to reach the former platform location (Figure 3E; ANOVA *p* = 0.003) than wild-type animals, independent of diet, again reflecting impaired spatial memory. Dietary intervention with DEU and FC had no effect on these parameters of spatial memory (ANOVA, *p*>0.05). Overall statistical analysis indicated significant genotype×diet interactions for the swim distance (*p* = 0.004) and mean velocity (*p* = 0.004).

Dietary intake affected the swim distance (ANOVA, *p* = 0.014) and mean velocity (ANOVA, *p* = 0.015) in AβPP-PS1 mice, such that AβPP-PS1 mice on the FC diet displayed a shorter swim distance (Figure 3F; post hoc *p* = 0.013) and lower mean swim velocity than the AβPP-PS1 animals on CO diet (post hoc, *p* = 0.015; AβPP-PS1-CO diet 17.3±0.4 cm/s, AβPP-PS1-FC diet 13.8±1.2 cm/s), and compared to the wild-type animals on FC diet (ANOVA, *p* = 0.009; wild-type-FC diet 16.9±0.4 cm/s), but not compared to the AβPP-PS1 animals on DEU diet (post hoc, *p* = 0.079; AβPP-PS1-DEU diet 16.8±0.9 cm/s).

#### Reverse Morris water maze (rMWM)

In the reverse Morris water maze (rMWM), mice have to learn to find a novel position for the hidden platform. This task is considered to be a test for new learning abilities, in which a previous successful strategy must be inhibited and a new strategy should be developed.

**Figure 4:**
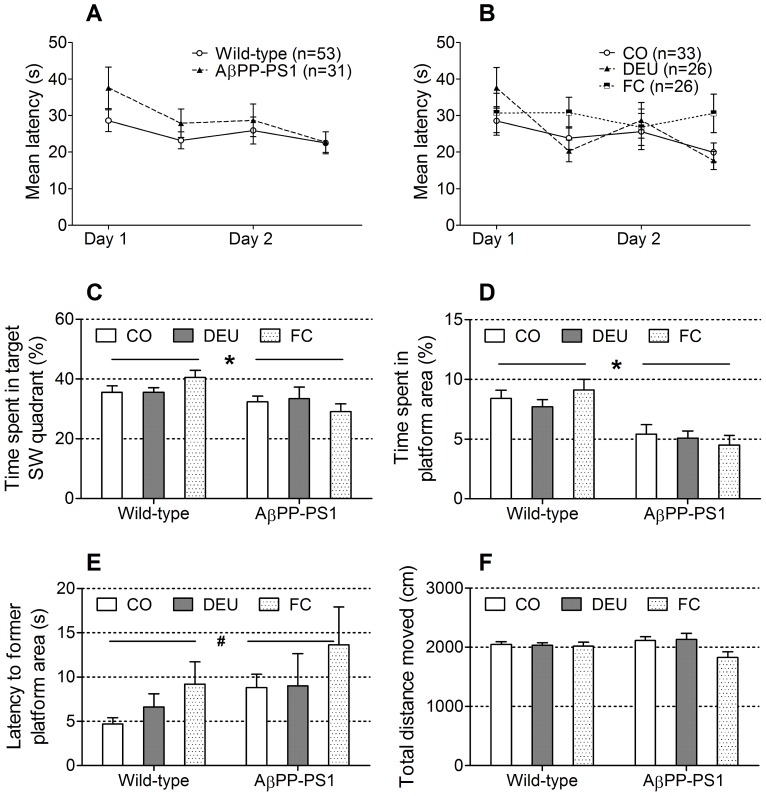
Reverse Morris water maze in 11-month-old AβPP-PS1 and wild-type mice on control and multi-nutrient diets. Spatial learning with an extra episodic memory component was measured in a 2-day acquisition phase, by determining the latency to find a hidden platform in the SW quadrant. Spatial memory was tested in the probe phase in which the percentage of time spent in the target SW quadrant, the time spent in the former platform area, the latency to the former platform area and the total distance moved were measured. A: Both AβPP-PS1 and wild-type mice showed a decrease in latency during training, independent of dietary intake. Overall escape latencies did not differ between AβPP-PS1 and wild-type mice. B: Animals on control (CO), the DHA, EPA and UMP (DEU) and Fortasyn® Connect (FC) diets showed a similar decrease in latency during training, independent of genotype. Overall escape latencies were unaffected by dietary intake. C: AβPP-PS1 mice spent less time in the target SW quadrant than wild-type mice, independent of dietary intake (*p = 0.006), although all animals performed well above 25% chance level. Time spent in the SW quadrant was unaffected by dietary intake. D: AβPP-PS1 mice spent less time in the former platform area than wild-type mice (*p<0.001), independent of dietary intake. Time spent in the exact former platform area was unaffected by dietary intake. E: AβPP-PS1 mice had a slightly higher latency to reach the former platform location than wild-type mice (^#^ *p* = 0.054), although this did not reach statistical significance. Latency to reach the former platform location was unaffected by dietary intake. F: No differences were observed between AβPP-PS1 and wild-type animals in the swim distance. Overall statistical analysis indicated a significant diet effect on the swim distance (*p* = 0.030). However, Tukey's post hoc analysis revealed no significant differences between any of the diet groups.

Both AβPP-PS1 and wild-type mice showed a decrease in escape latency during training (Figure 4A; ANOVA *p* = 0.016). No significant time×genotype, time×diet or time×genotype×diet interactions were observed (*p*>0.05), indicating that all animals learned the new platform position equally well. Escape latencies did not differ between AβPP-PS1 and wild-type mice (Figure 4A; ANOVA *p* = 0.160), nor did they differ between the animals on CO diet, DEU diet and FC diet (Figure 4B; ANOVA *p* = 0.274). No genotype×diet interaction was observed for the escape latencies (*p* = 0.922).

During acquisition training with a new platform location, swim speed decreased in both AβPP-PS1 and wild-type mice over time (data not shown; ANOVA *p*<0.001). No significant time×genotype, time×diet or time×genotype×diet interactions were observed (*p*>0.05). However, the average swim speed was significantly higher in AβPP-PS1 mice compared to wild-type animals during revere MWM training (ANOVA *p* = 0.008; wild-type 5.7±0.2 cm/s; AβPP-PS1 6.8±0.4 cm/s), independent of dietary intake (genotype×diet interaction *p* = 0.827). The mean swim speed did not differ (ANOVA, *p* = 0.511) between the animals fed the CO diet (5.9±0.3 cm/s), DEU diet (6.5±0.4 cm/s) or FC diet (6.0±0.4 cm/s).

During the probe trail, AβPP-PS1 mice spent less time in the target SW quadrant (Figure 4C; ANOVA *p* = 0.006) than wild-type mice, independent of diet (genotype×diet interaction, *p* = 0.120), although all animals performed well above 25% chance level, indicating good memorization of the platform quadrant. However, AβPP-PS1 mice also spent less time in the exact area where the platform had been located after relocation (Figure 4D; ANOVA *p*<0.001), indicating impaired spatial memory. Furthermore, they displayed a slightly higher latency to reach the former platform location than wild-type animals (Figure 4E; ANOVA *p* = 0.054), although this did not reach statistical significance. Dietary intervention with DEU and FC had no effect on these parameters of spatial memory (ANOVA, *p*>0.05). No differences were observed between AβPP-PS1 and wild-type animals in the swim distance (Figure 4F; ANOVA *p* = 0.909) or in the mean swim velocity (ANOVA *p* = 0.918; wild-type 16.3±0.2 cm/s, AβPP-PS1 16.2±0.4 cm/s). Overall statistical analysis indicated a significant effect of diet on swim distance (ANOVA, *p* = 0.030) and mean swim velocity (ANOVA *p* = 0.031; CO diet 16.6±0.3 cm/s, DEU diet 16.5±0.3 cm/s, FC diet 15.6±0.5 cm/s). However, Tukey's post hoc analysis revealed no significant differences between any of the diet groups (post hoc, *p*>0.1).

### Magnetic resonance imaging (MRI)

#### Magnetic resonance spectroscopy (MRS)

**Figure 5:**
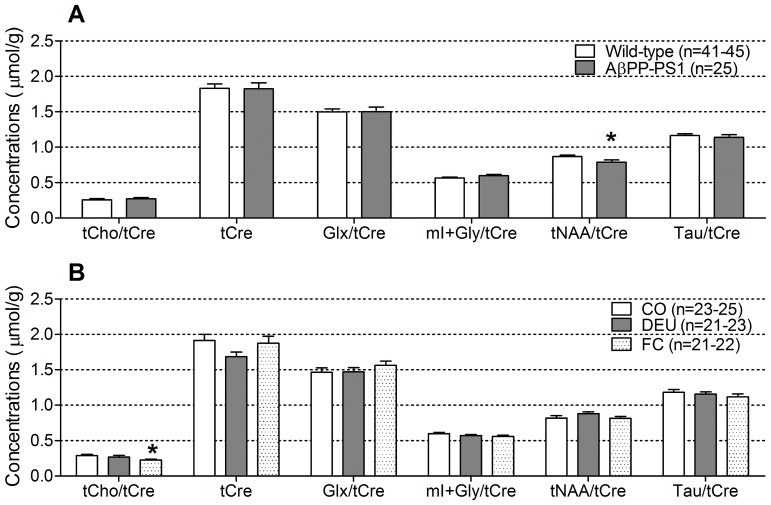
Hippocampal neurochemical profile of 12-month-old AβPP-PS1 and wild-type mice on control and specific multi-nutrient diets. The neurochemical profile of the hippocampus was determined with single voxel ^1^H MRS at 11.7 Tesla. A: AβPP-PS1 mice showed a significant decrease in tNAA/tCre compared to wild-type mice (*p = 0.030), independent of dietary intake. AβPP-PS1 and wild-type mice displayed similar levels of tCho/tCre, tCre, Glx/tCre, *m*I+Gly/tCre and Tau/tCre. B: Both wild-type and AβPP-PS1 mice fed the Fortasyn® Connect (FC) diet showed a significant decrease in tCho/tCre levels compared to animals fed the control (CO) diet (*p = 0.039), but no difference compared to mice fed the DHA, EPA and UMP (DEU) diet. Dietary intake did not affect the levels of tCre, Glx/tCre, *m*I+Gly/tCre, tNAA/tCre and Tau/tCre. Values represent the mean and SEM. tCho = choline-containing compounds; tCre = creatine and phosphocreatine; Glx = glutamine and glutamate; *m*I+Gly = *myo*-Inositol and glycine; tNAA = *N*-acetylaspartate and *N*-acetylaspartylglutamate; Tau = taurine.

To determine hippocampal metabolite concentrations, single voxel ^1^H MRS at 11.7 T was used (Figure 5). No differences were observed between wild-type and AβPP-PS1 mice (Figure 5A), nor between the different diet groups (Figure 5B) in the levels of creatine and phosphocreatine (tCre; ANOVA *p*>0.05). Therefore, tissue concentrations of metabolites are given relative to tCre as applied by others [71], [72].

AβPP-PS1 mice had significantly lower levels of *N*-acetylaspartate and N-acetylaspartylglutamate (tNAA; ANOVA *p* = 0.030) than wild-type mice, independent of diet (genotype×diet interaction, *p* = 0.513), indicating decreased neuronal integrity (Figure 5A). Wild-type and AβPP-PS1 mice had similar levels of choline-containing compounds (tCho; ANOVA *p* = 0.079), glutamine and glutamate (Glx; ANVOA *p* = 0.963), *myo*-Inositol and glycine (*m*I+Gly; ANOVA *p* = 0.196) and taurine (Tau; ANOVA *p* = 0.475).

Statistical analysis indicated a significant effect of dietary intake on tCho levels, independent of genotype (Figure 5B; ANOVA *p* = 0.035). Post hoc analysis revealed that animals fed the FC diet had significant lower tCho levels than animals fed the CO diet (post hoc, *p* = 0.039), but no difference compared to the animals fed the DEU diet (post hoc, *p* = 0.228). No differences were observed between the different diets in relative concentrations of Glx (ANOVA, *p* = 0.627), mI+Gly (ANOVA, *p* = 0.400), tNAA (ANOVA, *p* = 0.156) and Tau (ANOVA, *p* = 0.523).

### Immunohistochemistry

#### Doublecortin staining

Immature neurons were visualized with a polyclonal antibody against doublecortin. Doublecortin-positive (Dcx+) cells were counted in three alternating hippocampal sections as a measure for neurogenesis.

**Figure 6:**
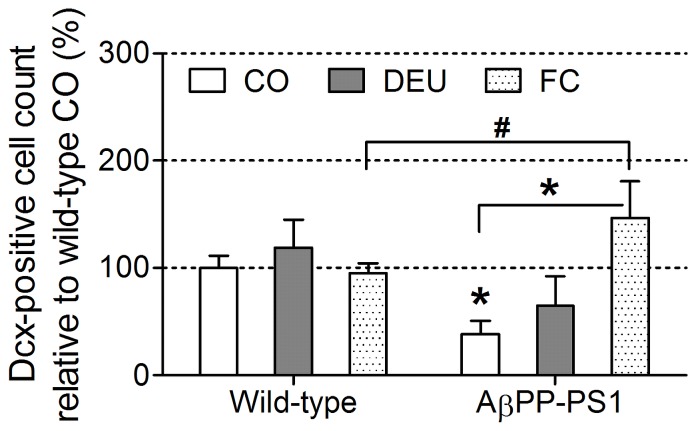
Neurogenesis in 12-month-old AβPP-PS1 and wild-type mice on control and specific multi-nutrient diets. From 2 months of age, mice were fed either a control (CO), a DHA, EPA and UMP (DEU) or a Fortasyn® Connect (FC) diet. The amount of immature neurons in the subgranular zone of the hippocampus were visualized immunohistochemically with a polyclonal goat anti-doublecortin antibody (1:3000) as a measure for neurogenesis. Values represent the mean and SEM and are relative (%) compared to wild-type mice on CO diet. In wild-type animals, the relative amount of doublecortin-positive (Dcx+) immature neurons was similar for all diet groups. However, in AβPP-PS1 mice the relative amount of Dcx+ immature neurons was affected by dietary intake (*p* = 0.017). Post hoc analysis revealed that AβPP-PS1 mice fed the CO diet displayed a significantly decreased relative amount of Dcx+ immature neurons compared to wild-type mice on the CO diet (*p = 0.004). The FC diet significantly increased the relative amount of Dcx+ immature neurons in AβPP-PS1 mice as compared to AβPP-PS1 mice on CO diet (*p = 0.015). Furthermore, AβPP-PS1 mice on the FC diet had slightly higher relative amount of Dcx+ immature neurons than wild-type animals on the FC diet (^#^ *p* = 0.053), although this did not reach statistical significance.

Overall statistical analysis indicated a significant genotype×diet interaction (ANOVA, *p* = 0.004) for the relative amount of Dcx+ cells. In wild-type animals, the relative amount of Dcx+ cells were similar for all diet groups (ANOVA, *p* = 0.530). However, in AβPP-PS1 mice, the relative amount of Dcx+ cells was affected by dietary intake (ANOVA, *p* = 0.017). Post hoc analysis revealed that AβPP-PS1 mice on CO diet showed a decreased amount of Dcx+ cells compared to wild-type mice on CO diet (Figure 6; ANOVA, *p* = 0.004), indicating decreased neurogenesis in AβPP-PS1 mice on a normal control diet. Moreover, AβPP-PS1 mice fed the FC diet had a significantly higher relative amount of Dcx+ cells than AβPP-PS1 animals fed the CO diet (post hoc, *p* = 0.015), suggesting that the FC diet restored neurogenesis in AβPP-PS1 mice. Furthermore, AβPP-PS1 mice on FC diet also had a slightly higher relative amount of Dcx+ cells than wild-type animals on FC diet (ANOVA, *p* = 0.053), although this did not reach statistical significance.

### Biochemical analyses

#### Serum and brain sterol analysis

**Figure 7:**
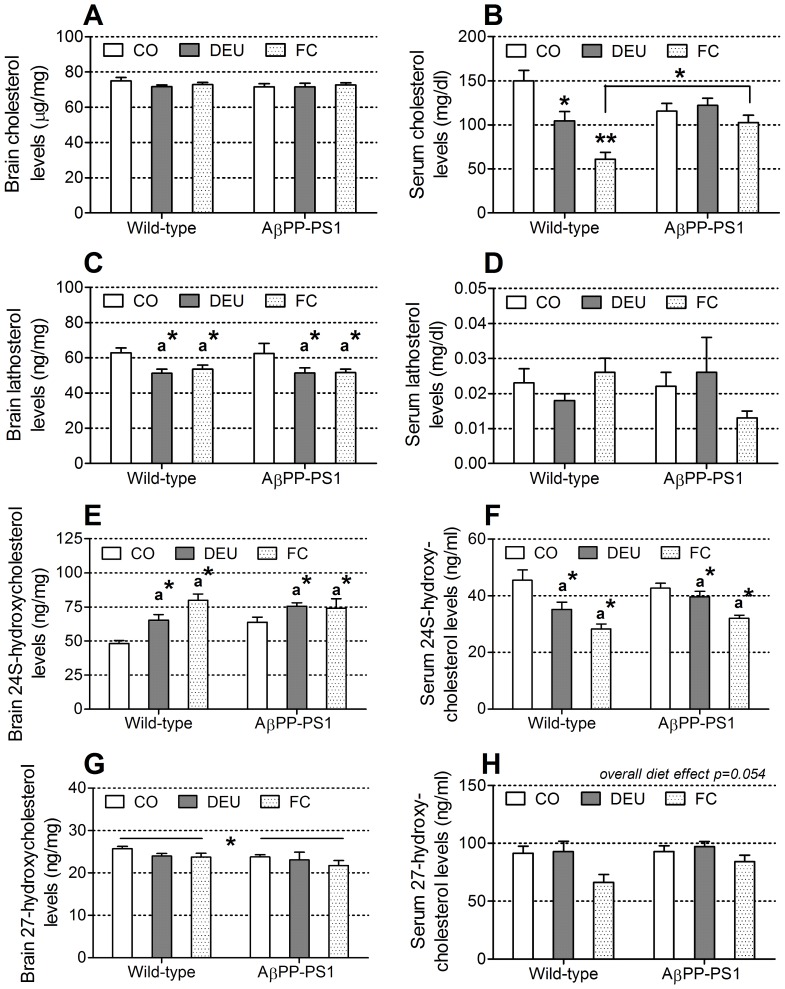
Sterol levels in 12-month-old AβPP-PS1 and wild-type mice on control and specific multi-nutrient diets. Sterol levels were determined in brain homogenates and blood serum by gas-chromatography-mass-spectrometry-selected ionmonitoring. A–B: Cholesterol levels were similar in the brains of AβPP-PS1 and wild-type mice and were unaffected by dietary intake. In blood sera of wild-type mice, the Fortasyn® Connect (FC) diet decreased cholesterol levels as compared to the control (CO) and the DHA, EPA and UMP (DEU) diets (**), whereas the DEU diet led to intermediate cholesterol levels as compared to the CO and FC diets. Only wild-type mice on FC diet displayed decreased levels of serum cholesterol compared to AβPP-PS1 mice, *p<0.05. C-D: Lathosterol levels were similar in the brains and blood sera of AβPP-PS1 and wild-type mice. Serum lathosterol levels were unaffected by dietary intake, but brain lathosterol levels were decreased in AβPP-PS1 and wild-type mice on the DEU and FC diets as compared to the CO diet (a* *p*<0.05). E–F: 24-hydroxycholesterol levels were similar in the brains and blood sera of AβPP-PS1 and wild-type mice. Brain 24-hydroxycholesterol levels were increased in AβPP-PS1 and wild-type mice on the DEU and FC diets as compared to the CO diet (a*), whereas serum 24-hydroxycholesterol levels were decreased in AβPP-PS1 and wild-type mice on the DEU and FC diets as compared to the CO diet (a*), a*p<0.05. G–H: Brain 27-hydroxycholesterol levels were decreased in AβPP-PS1 mice compared to wild-type mice (*p = 0.040), but were unaffected by dietary intake. Serum 27-hydroxycholesterol levels were similar between AβPP-PS1 and wild-type mice, independent of dietary intake. Overall analysis revealed a tendency for an effect of dietary intake (*p* = 0.054), but no post hoc analyses were performed since it did not reach statistical significance.

Brain cholesterol levels (Figure 7A) were similar between wild-type and AβPP-PS1 mice (ANOVA, *p* = 0.369), and between the diet groups (ANOVA, *p* = 0.637). However, dietary intervention significantly affected serum cholesterol levels (Figure 7B) in wild-type mice (ANOVA, *p*<0.001), but not in AβPP-PS1 mice (ANOVA, *p* = 0.314), as indicated by a significant genotype×diet interaction (*p* = 0.009). Post hoc analysis revealed that in wild-type mice the DEU diet decreased serum cholesterol levels compared to the CO diet (post hoc, *p* = 0.014), and the FC diet decreased serum cholesterol levels further (post hoc, *p*<0.001 compared to CO diet and *p* = 0.026 compared to DEU diet). No genotype effects on serum cholesterol levels were observed between the wild-type and AβPP-PS1 mice on CO (ANOVA, *p* = 0.101) and DEU diets (ANOVA, p = 0.353), but AβPP-PS1 mice on FC diet had significantly higher serum cholesterol levels than wild-type mice on FC diet (ANOVA, *p* = 0.007).

In addition, brain levels of lathosterol (Figure 7C), a main cholesterol precursor in the “de novo” synthesis pathway, were similar between wild-type and AβPP-PS1 mice (ANOVA, *p* = 0.800). Dietary intake did affect the brain levels of lathosterol (ANOVA, *p* = 0.003), such that animals fed the DEU (post hoc, *p* = 0.003) and FC diets (post hoc, *p* = 0.010) displayed significantly decreased levels of brain lathosterol compared to animals fed the CO diet, independent of genotype (genotype×diet interaction, *p* = 0.888). Serum lathosterol levels (Figure 7D) were similar between wild-type and AβPP-PS1 mice (ANOVA, *p* = 0.609), and between the diet groups (ANOVA, *p* = 0.709).

Furthermore, the levels of cholesterol's brain specific oxidative metabolite 24S-hydroxycholesterol did not differ significantly in the brains (ANOVA, *p* = 0.072) and sera (ANOVA, *p* = 0.510) of wild-type and AβPP-PS1 mice. Dietary intake did affect both brain (ANOVA, *p*<0.001) and serum (ANOVA, *p* = 0.001) 24S-hydroxycholesterol levels, such that animals fed the DEU (post hoc, *p* = 0.004) and FC diets (post hoc, *p*<0.001) had significantly higher levels of 24S-hydroxycholesterol in the brain compared to animals fed the CO diet (Figure 7E), independent of genotype (genotype×diet interaction, *p* = 0.054), indicating increased conversion of cholesterol in the brain due to dietary intervention. In contrast, serum 24S-hydroxycholesterol levels (Figure 7F) were decreased in animals fed the DEU (post hoc, *p* = 0.028) and FC diets (post hoc, *p*<0.001) compared to animals fed the CO diet, independent of genotype (genotype×diet interaction, *p* = 0.477).

Another oxidative metabolite of cholesterol, 27-hydroxycholesterol (Figure 7G), was significantly decreased in the brains of AβPP-PS1 mice compared to wild-type animals (ANOVA, *p* = 0.040), independent of diet (genotype×diet interaction, *p* = 0.817), suggesting a decreased flux of cholesterol from the periphery into the brain [73]–[75]. No significant differences could be observed in the levels of brain 27-hydroxycholesterol between the diet groups (ANOVA, *p* = 0.073). Serum 27-hydroxycholesterol levels (Figure 7H) were similar between wild-type and AβPP-PS1 mice (ANOVA, *p* = 0.256). Overall analysis revealed a tendency for an effect of diet intervention (ANOVA, *p* = 0.054) on serum 27-hydroxycholesterol levels, but this did not reach statistical significance.

### Brain fatty acid analysis

**Figure 8:**
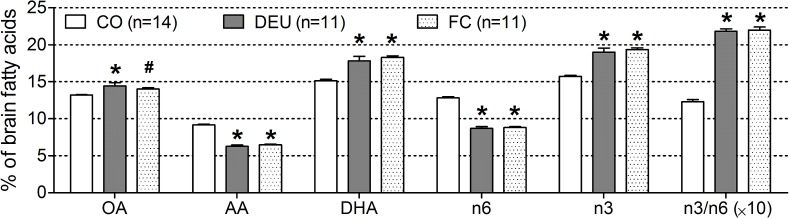
Brain fatty acids in 12-month-old AβPP-PS1 and wild-type mice on control and specific multi-nutrient diets. The relative concentrations of different fatty acids were determined in the lipid fraction of brain homogenates, and were similar between AβPP-PS1 and wild-type mice. Dietary intake affected the relative concentrations of different fatty acids similarly in AβPP-PS1 and wild-type mice. Both the DHA, EPA and UMP (DEU) diet and the Fortasyn® Connect (FC) diet increased the relative concentrations of omega-3 fatty acids (n3) and decreased the relative concentrations of omega-6 fatty acids (n6), resulting in a pronounced shift in n3/n6 ratio in favor of the n3 fatty acids as compared to the control(CO) diet, *p<0.001. The reduction of the relative n6 content was mainly caused by a decrease in arachidonic acid (AA; C20∶4n6), while the higher n3 content was mainly caused by an increase in docosahexaenoic acid (DHA; C22∶6n3), *p<0.001. The relative concentration of oleic acid (OA; C18∶1n9) was increased due to intake of the DEU diet *p = 0.005), while it was only slightly, but not significantly, increased due to intake of the FC diet (^#^ *p* = 0.066). The relative amount of palmitic acid (C16∶0); stearic acid (C18∶0), saturated fatty acids, mono-unsaturated fatty acids and poly-unsaturated fatty acids were unaffected by dietary intake (data not shown).

Dietary intake affected the relative concentrations of oleic acid (ANOVA, *p* = 0.011), arachidonic acid (ANOVA, *p*<0.001), docosahexaenoic acid (ANOVA, *p*<0.001), omega-3 fatty acids (n3 FAs; ANOVA p<0.001), and omega-6 FAs (ANOVA; p<0.001) in both wild-type and AβPP-PS1 mice (Figure 8). Animals fed the DEU and FC diets (post hoc *p*<0.001) showed a shift in the balance between n3 and n6 fatty acids as compared to animals fed the CO diet (main ANOVA diet effect, *p*<0.001). The relative amount of n3 FA in DEU and FC fed mice increased compared to the CO diet (post hoc, *p*<0.001), whereas the relative amount of n6 decreased (post hoc, *p*<0.001), resulting in a pronounced shift in the n3/n6 ratio in DEU and FC fed mice in favor of the n3 FA. The reduction of the relative n6 content was mainly caused by a relative decrease in arachidonic acid (AA; C20∶4n6; post hoc *p*<0.001), while the higher n3 content originated from a relative increase in DHA (C22∶6n3; post hoc *p*<0.001) in both the DEU and FC fed animals as compared to the CO fed animals. Furthermore, the relative concentration of oleic acid (OA; C18∶1n9) was significantly increased in the DEU fed mice (post hoc, *p* = 0.005), and also slightly increased in the FC fed animals (post hoc, *p* = 0.066) as compared to the CO diet, although it did not reach statistical significance.

No significant differences were observed between the DEU and FC diets in any fatty acid analyzed (post hoc, *p*>0.05). The relative amounts of palmitic acid (C16∶0; ANOVA *p* = 0.714), stearic acid (C18∶0; ANOVA *p* = 0.841), total saturated fatty acids (SFA; ANOVA *p* = 0.793), total mono-unsaturated fatty acids (MUFA; ANOVA *p* = 0.316) and total poly-unsaturated fatty acids (PUFA; ANOVA *p* = 0.459) were unaffected by the diets (data not shown). No significant genotype effects or genotype×diet interactions were found for any of the fatty acids analyzed (ANOVA, *p*>0.05).

#### Quantitative real-time PCR (qRT-PCR)

**Figure 9:**
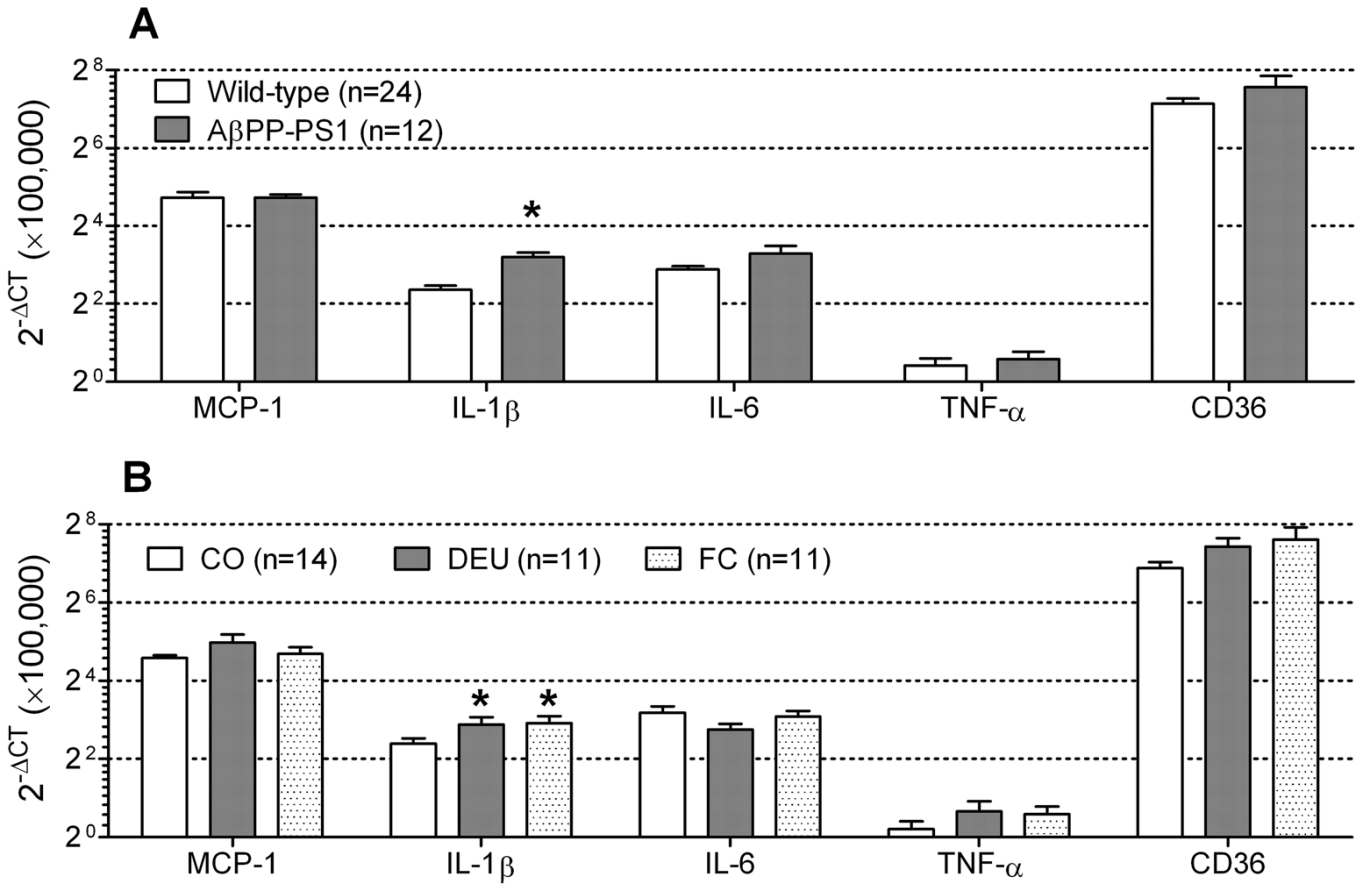
Inflammatory markers in 12-month-old AβPP-PS1 and wild-type mice on control and specific multi-nutrient diets. Inflammatory markers were determined by quantitative real-time PCR in a part of the snap-frozen brain tissue, which included both hippocampi, using primers for monocyte chemoattractant protein 1 (MCP-1), interleukin-1β (IL-1β), interleukin-6 (IL-6), tumor necrosis factor-α (TNF-α), cluster of differentiation 36 (CD36), and glyceralehyde-3-phospate dehydrogenase (GADPH). Relative gene expression ratios were calculated according to the comparative C_T_ method (2^−ΔCT^ method), by subtracting the C_T_ value of the housekeeping gene GADPH from the C_T_ values of the inflammatory markers(ΔC_T_). A: AβPP-PS1 and wild-type mice displayed similar levels of MCP-1, IL-6, TNF-α and CD36. However, IL-1β levels were increased in AβPP-PS1 mice compared to wild-type animals, independent of dietary intake (*p<0.001). B: Levels of MCP-1, IL-6, TNF-α and CD36 were unaffected by dietary intake. However, the levels of IL-1β were affected by dietary intake, independent of genotype (*p* = 0.005). Both the DHA, EPA and UMP (DEU) diet (*p* = 0.034) and the Fortasyn®Connect (FC) diet (*p* = 0.013) increased the levels of IL-1β as compared to the control (CO) diet in AβPP-PS1 and wild-type mice.

Wild-type and AβPP-PS1 mice displayed similar levels of MCP-1 (ANOVA, *p* = 0.927), IL-6 (ANOVA, *p* = 0.081), TNF-α (ANOVA, *p* = 0.701) and CD36 (ANOVA, *p* = 0.180). However, IL-1β levels were significantly increased in AβPP-PS1 mice compared to wild-type animals (Figure 9A; ANOVA, *p*<0.001), independent of dietary intake (genotype×diet interaction, *p* = 0.272). Furthermore, overall statistical analysis indicated a significant effect of dietary intake on the levels of IL-1β (ANOVA, *p* = 0.005). Post hoc analysis revealed that both wild-type and AβPP-PS1 animals fed the DEU (post hoc, *p* = 0.034) and FC diets (post hoc, *p* = 0.013) had significantly higher levels of IL-1β than animals fed the CO diet (Figure 9B). No differences were observed between the different diets in the levels of MCP-1 (ANOVA, *p* = 0.349), IL-6 (ANOVA, *p* = 0.232), TNF-α (ANOVA, *p* = 0.707) and CD36 (ANOVA, *p* = 0.157).

## Discussion

In the present study, we investigated the extent to which long-term consumption of two specific multi-nutrient enriched diets, developed to support membrane synthesis and maintenance, can modulate behavior, cognition, hippocampal metabolite levels, neurogenesis and inflammation in 11-12-month-old AβPP-PS1 mice. An overview of the main results found in the current study is shown in Table 3.

**Table 3:** Overview of the main genotype and diet effects found in the current study.

Effects of genotype:		Compared to wild-type mice
		AβPP-PS1 mice
Open field	Behavior & activity	Increased activity & anxiety-related behavior
		Decreased exploration
Morris water maze	Learning & memory	Decreased learning & memory
reverse MWM	Learning & memory	Decreased memory
Hippocampal 1H MRS	Metabolite levels	Decreased tNAA/tCre
Immature neurons	Hippocampus	Decreased neurogenesis
Sterol analyses	Serum	n.s.
	Brain	Decreased 27-hydroxycholesterol
Fatty acid analysis	Brain	n.s.
Inflammatory markers	Brain	Increased IL-1β

Effects of specific multi-nutrient diets:		Compared to littermates on control (CO) diet	
		DEU diet	FC diet
Open field	Behavior & activity	Increased activity	n.s.
		Increased anxiety	Decreased anxiety
Morris water maze	Learning & memory	n.s.	n.s.
reverse MWM	Learning & memory	n.s.	n.s.
Hippocampal 1H MRS	Metabolite levels	n.s.	Decreased tCho/tCre
Immature neurons	Hippocampus	n.s.	Increased
	*effect in AβPP-PS1 only		neurogenesis*
Sterol analyses	Serum	Decreased cholesterol*	Decreased cholesterol*
	*effect in wild-type only	Decreased 24S-	Decreased 24S-
		hydroxycholesterol	hydroxycholesterol
	Brain	Decreased lathosterol	Decreased lathosterol
		Increased 24S-	Increased 24S-
		hydroxycholesterol	hydroxycholesterol
Fatty acid analysis	Brain	Increased n3 content	Increased n3 content
		Decreased n6 content	Decreased n6 content
		Increased OA content	Increased OA content #
Inflammatory markers	Brain	Increased IL-1β	Increased IL-1β
			

1 H MRS = proton magnetic resonance spectroscopy; tNAA = *N*-acetylaspartate and *N*-acetylaspartylglutamate; tCre = creatine and phosphocreatine; IL-1β = interleukin-1β; DEU diet = DHA, EPA, UMP diet; FC diet = Fortasyn® Connect diet; tCho = choline-containing compounds; n3 = omega-3 fatty acids; n6 = omega-6 fatty acids; OA = oleic acid (18∶1n9); n.s. = not significant; # = tendency (0.5>*p*<0.7).

In agreement with previous results from our lab [58], [76], 11-month-old AβPP-PS1 mice displayed increased activity and anxiety-related behavior, and decreased explorative behavior in the open field as compared to age-matched wild-type mice. Hyperactivity and decreased explorative behavior are specific characteristics of many AβPP transgenic mice [77]–[79] and may be explained as a result of elevated anxiety levels [80], [81]. Curiosity motivates mice to explore a novel environment, but this exploratory drive is in conflict with fear of the unknown. Hyperactivity and anxiety-related behavior in AβPP transgenic mice resemble anxiety symptoms and restlessness, which occur in up to 70% of AD patients during the course of their illness and are significantly correlated with impairments in activities of daily living [82]–[84]. Dietary intervention with the DEU and FC diets differentially affected open field behavior in both AβPP-PS1 and wild-type mice. While the DEU diet increased general locomotor activity (restlessness) and anxiety-related exploration (e.g. wall leaning), our results suggest that the FC diet could have an anxiolytic effect, since it decreased the time spent in the corners of the open field as compared to the animals on CO and DEU diets. Previous studies have shown that rats fed an n3 lc-PUFA deficient diet display increased anxiety-related behavior in the open field and elevated plus maze tasks as compared to animals fed an n3 lc-PUFA adequate diet [85], [86]. Supplementation with n3 lc-PUFAs led to substantial reduction in the anxiety levels of n3 lc-PUFA deficient rats [86],[87] and mice [88]. Moreover, n3 fatty acid deficiency has been linked to increased vulnerability to stress, elevated aggression, and increased depressive-like symptoms in rodents [89]. Supplementation with n3 lc-PUFAs has also shown beneficial effects on depressive symptoms and agitation in patients with mild to moderate AD [90]. However, it should be noted that both the DEU diet and the FC diet contained equal amounts of n3 lc-PUFAs, suggesting that the combination of additional nutrients in the FC diet was important for the efficacy on anxiety-related behavior. Interestingly, Schipper et al. showed that a combination of n3 lc-PUFAs, phospholipids and B-vitamins (which are also part of the current FC diet) completely abolished anxiety-related behavioral responses, increased social behavior and facilitated fear extinction recall in serotonin transporter knockout (SERT-ko) rats [91]. Furthermore, the FC diet also reduced anxiety-related behavior in the open field in 12-month-old apolipoprotein E (apoE)-ε4/ε4 and apoE knockout mice [92]. Combined, these results suggest that supplementation with the FC diet could potentially be beneficial for several neurodegenerative and neurological disorders in which patients exhibit symptoms of agitation, anxiety and depression.

In line with our previous results [58], [76], 11-month-old AβPP-PS1 mice also showed impaired performance in the MWM and rMWM as compared to age-matched wild-type animals. Although both AβPP-PS1 and wild-type mice showed a similar decrease in escape latency during training in the MWM, overall escape latencies were significantly higher in AβPP-PS1 mice, independent of dietary intake. Longer escape latencies during spatial navigation can be caused by slower swim speed, although this is not a confounding factor in the current study, since AβPP-PS1 mice displayed higher swim speeds during the task acquisition trials. Longer escape latencies during spatial navigation may also be caused by the use of certain, less efficient, search strategies, such as a constant random search of the entire surface area of the pool, which would indicate a complete lack of spatial learning abilities, or by persistent performance of a less efficient than spatial (direct) search strategy, such as circling the pool at a certain distance from the wall to find the platform [93], [94]. In this strategy, mice have not used the spatial cues to learn the location of the platform, although such a strategy would result in a successful location of the escape platform during training. Since AβPP-PS1 mice searched the target NE quadrant during the probe trial at chance level (25% or less), our results might imply that the AβPP-PS1 mice made use of a random or a persistent non-spatial search strategy to locate the platform during acquisition training. This is in line with a study by O′Leary and Brown, in which the search strategies used by 16-month-old AβPP-PS1and wild-type mice during visuo-spatial navigation in the Barnes Maze were analyzed [95]. 16-month-old AβPP-PS1 mice predominantly made use of a random search strategy to locate the escape hole in the Barnes Maze, whereas wild-type mice predominantly made use of a spatial (direct and accurate) search strategy. Similar results have been found for the search strategies used by the TgCRND8 transgenic AβPP mouse model in the MWM test [96], [97]. Moreover, it was shown that C57BL6 mice, treated with Temozolomide (TMZ) to suppress adult hippocampal neurogenesis, displayed a delayed (or even absent) use of directed and place specific search patterns in the (reversal) MWM test compared to untreated mice, suggesting that hippocampal neurogenesis is necessary for adding flexibility to some hippocampus-dependent qualitative parameters of learning [93]. Consistent with our current findings, reduced hippocampal neurogenesis has been found previously in AβPP-PS1 mice [98]–[100] and in AD patients [101], [102], and might underlie some aspects of the cognitive deficits in AD.

In line with our previous findings [76], AβPP-PS1 and wild-type mice showed a similar decrease in escape latency during training in the reversal MWM. The reversal task requires selective memory retrieval of the newly learned location of the platform, and contains therefore an extra episodic component [60], [103]. Since episodic memory impairment is a major characteristic in early AD [104]–[106], our results in AβPP-PS1 mice resemble the problems that are present in early AD patients. Noteworthy are the relatively low escape latencies of both AβPP-PS1 and wild-type mice during the initial trials of the rMWM acquisition training. Although wild-type mice displayed good short-term spatial memory for the NE platform location during the probe trial of the MWM, it appears that this information might not have been sufficiently consolidated in the long-term memory since they displayed more “random” search behavior during the initial rMWM trials (resulting in lower escape latencies) than expected if animals had good long-term memory for the former platform location. One possible explanation could be that the MWM task as used in the current study was slightly too difficult to optimally master within 4 days of training. For mice, a MWM pool with a diameter of 120 cm and a platform of 10–12 cm in diameter (or even larger) is commonly used, resulting in a search area to target size of 144∶1 to 100∶1 (or even lower). In the current study, we used a pool with a diameter of 104 cm and a platform of 8 cm in diameter, resulting in a search area to target size of 169∶1, thereby increasing the MWM task difficulty [107], [108].

Previous studies have shown that n3 lc-PUFA intake may improve cognition in both mice and rats [58], [109], [110]. Besides n3 lc-PUFAs, other nutrients can also affect spatial memory performance. Supplementing CDP-choline or UMP and choline to rats improved spatial memory in the water maze [111], [112]. In addition, transgenic mice fed vitamin E [113] or vitamin B3 [114] showed normalized escape latency in the water maze. Furthermore, a combination of vitamin E and folic acid prevented human Aβ_1-40_-induced impairment of water maze learning in 3-month-old male *Swiss* mice [115]. However, in agreement with our current results, there are also some reports showing no effect of n3 lc-PUFA intake on learning and memory performance [89], [116], [117]. Moreover, most intervention studies based on single nutrient supplementation with n3 lc-PUFAs, B-vitamins or vitamin E have failed to show any protective effect on AD [39]–[41], [118] or cognitive decline [119]–[121], except in some patients with mild AD [122], [123]. Interestingly, clinical studies with the multi-nutrient FC supplementation have shown beneficial effects on memory performance in patients with mild AD [51]–[53], although we could not replicate these findings in our AβPP-PS1 mouse model when using the standard conventional measures of performance in the MWM. However, the FC diet did recently improve the search strategy of AβPP-PS1 mice in the acquisition of the MWM, by switching from predominantly random search strategies towards a more efficient search strategy, suggesting some beneficial effects of the FC diet on the ability to cope with cognitive impairment in the AβPP-PS1 mouse model [124].

^1^H MR spectroscopy revealed decreased tNAA/tCre levels in the hippocampus of 12-month-old AβPP-PS1 mice as compared to wild-type mice, in accordance with our previous results [76]. The reduction of tNAA levels is one of the most consistent findings using ^1^H MRS in AD patients [125]–[128] and in several transgenic AβPP animals models for AD [129]–[133], and is commonly interpreted as a result of neuronal dysfunction or neuronal loss [134], [135]. Furthermore, many previous ^1^H MRS studies have found elevated levels of *myo*-Inositol (*m*I) in the temporal, parietal and occipital lobes of AD patients [126], [128], which has been associated with enhanced inflammatory processes. Reports on *m*I levels in transgenic animals models are highly inconsistent, since disturbances in *m*I levels were found to occur at different ages in different transgenic species apparently depending on the interplay of mouse strain, transgene and disease progression [136]. For example, the AβPPswe (Tg2576) model and the AβPP-PS2N1411 (PS2APP) model do not show any change in *m*I levels throughout life compared to age-matched wild-type mice, even though these animal models display decreased NAA levels in the frontal cortex at 19–24 months of age, when Aβ deposits are widespread [129], [137]. In the case of the AβPPswe-PS1M146L model, one study reported the most profound increase in *m*I levels after 20 months of age [130], whereas another study reported a decrease in *m*I levels at 2.5 months of age as compared to age-matched wild-type mice [131]. In the current and in our previous study [76], we did not observe any differences in the levels of *m*I+Gly between AβPP-PS1 and wild-type mice. In contrast, Chen and colleagues observed significantly increased levels of *m*I in 3-, 5- and 8-month-old AβPPswe-PS1dE9 mice, when pathology showed activation and proliferation of astrocytes in the frontal cortex and hippocampus [138], [139]. The discrepancy between the results found by Chen et al. and our own findings are most likely due to differences in methodology, e.g. the field strength of the MR system, the acquisition parameters used, the exact position of the spectroscopic volume of interest, the age of the animals, and the amount of animals used.

Disturbances of several other metabolites have been found in AD patients as well, although the reports are inconsistent. Some studies identified elevated choline-containing compounds (tCho) and creatine (Cre) in AD patients [140]–[142], whereas others did not [125], [143]. One possible explanation for the elevation of tCho in AD is increased membrane turnover due to neurodegenerative processes [144], [145]. It has also been postulated that the elevation of the tCho peak is the consequence of membrane phosphatidylcholine catabolism in order to provide free choline for the chronically deficient acetylcholine production in AD [146], [147]. In agreement with our current findings, no differences were observed between AβPP-PS1 and wild-type mice at any age tested in the levels of tCho [76], [133], [138]. Dietary intake with the FC diet significantly decreased the levels of tCho in AβPP-PS1 and wild-type mice, suggesting diminished membrane turnover. Since the FC diet provides precursors and cofactors in membrane synthesis and maintenance that are not supplemented in the CO and DEU diets, such as choline chloride and soy lecithin, these additional nutrients might underlie the decreased levels of tCho. ^1^H MRS studies to investigate the effect of the multi-nutrient FC diet on brain metabolites in mild AD patients are currently ongoing.

Dietary intervention with the FC diet, but not with the DEU diet, restored neurogenesis in AβPP-PS1 mice. Reduced hippocampal neurogenesis has been found previously in AβPP-PS1 mice beyond 8 months of age [99], [100], [148] and in AD patients [102], [149], [150]. In line with our results, previous studies have shown that n3 lc-PUFAs [151], [152], folic acid [153], [154], and vitamins and antioxidants [155], [156] may promote neurogenesis. Since the FC diet did not affect neurogenesis in wild-type mice, our results could imply that this dietary intervention may especially be beneficial when neurogenesis is severely compromised. Dietary intervention with the FC diet might therefore be of interest for several other neurodegenerative and neurological disorders in which neurogenesis is impaired, such as Parkinson's disease and major depression.

Quantitative real-time PCR revealed increased IL-1β mRNA levels in 12-month-old AβPP-PS1 mice as compared to age-matched wild-type mice. In response to brain injury or infection, IL-1β is both expressed by and targeted to many different cell types within the brain, such as microglia, astrocytes, endothelial cells, infiltrating leukocytes, neurons and oligodendrocytes [157]–[159]. IL-1β has been implicated to be at or near the top of the cytokine signaling cascade that initiates the neuroinflammatory changes seen in AD (reviewed in [160], [161]). It has been suggested that the initial burst of IL-1β production might actually have a beneficial role in AD, by enhancing the microglial clearance of amyloid plaques [161]–[163]. However, chronic elevation of IL-1β is detrimental, since IL-1β is capable of propagating inflammatory responses by increasing its own expression [161], [162], [164] and inducing the expression of several other pro-inflammatory cytokines, such as TNF-α and IL-6 [165], and chemokines, such as MCP-1 [166], [167]. Our results of increased IL-1β mRNA levels in 12-month-old AβPP-PS1 mice, but no changes (yet) in the mRNA levels of MCP-1, IL-6, TNF-α and CD36, might reflect the initial burst of IL-1β production in response to brain injury or toxic agents such as Aβ.

Surprisingly, IL-1β mRNA levels were also elevated in both AβPP-PS1 and wild-type mice in response to dietary intervention with the DEU and FC diets. This seems contradictory to the anti-inflammatory and immunosuppressive properties ascribed to n3 lc-PUFAs, B vitamins and antioxidants [28], [34], [168]. However, IL-1β signaling has also been associated with neuroprotective mechanisms, including mediating the production of survival signals such as nerve growth factor [169], [170] and mediating re-myelination of the central nervous system by stimulating oligodendrocytes proliferation [159], [171]. Interestingly, results from our own lab indicate that both the DEU and FC diets prevent axonal disconnection and myelin degradation, determined by diffusion tensor MR imaging, in the 12-month-old AβPP-PS1 and wild-type mice used in the current study (Zerbi et al., submitted data). However, since prolonged elevation of IL-1β can initiate chronic neuroinflammatory processes, the question remains whether the effects of these multi-nutrient diets on IL-1β production are ultimately beneficial or detrimental in the AβPP-PS1 mouse model.

In conclusion, we showed that specific multi-nutrient diets can ameliorate some AD-related pathologies in 11-12-month-old AβPPswe-PS1dE9 mice. Although both diets were equally effective in changing brain fatty acid profiles and cholesterol metabolism, the diets differentially affected open field behavior, hippocampal metabolite levels and neurogenesis, suggesting that the effectiveness of specific nutrients may depend on the dietary context in which they are provided. The multi-nutrient enriched FC diet with DHA, EPA, UMP, phospholipids, choline, folic acid, vitamins B6, B12, C, E and selenium (Fortasyn® Connect; FC diet) was more effective than the DEU diet enriched with DHA, EPA and UMP in counteracting neurodegenerative aspects of AD and enhancing processes involved in neuronal maintenance and repair. Intervention with the FC diet might therefore be of interest for several other neurodegenerative and neurological disorders.
